# The *ʿarżuḥāl*s of Sylvester, Patriarch of Antioch: Negotiating church affairs with the sublime porte in the first half of the 18th Century

**DOI:** 10.12688/openreseurope.23367.1

**Published:** 2026-04-22

**Authors:** Radu Dipratu

**Affiliations:** 1 Institute for South-East European Studies, Bucharest, Romania

**Keywords:** Orthodox, Catholics, Ottoman Empire, non-Muslims, petitions, conversions.

## Abstract

While persecutions endured by non-Muslims under the “Turkish yoke” still represent a common trope both in the public perception and in certain academic circles in Orthodox-majority countries today, recent scholarly work has offered more nuanced approaches to the history of the Orthodox Churches under Ottoman rule. Making use of the rich information provided by Ottoman archival sources, this paper examines several unpublished documents from the Ottoman Archives in Istanbul, representing petitions ( *ʿarżuḥāl* ) submitted by Sylvester, the Orthodox Patriarch of Antioch (1724–1766). It is no surprise that an important theme of these petitions concerned the conflict between the Orthodox and Catholic factions in Greater Syria, the latter being recently energised by the election of a separate patriarch in 1724, in the person of Cyril VI, and the establishment of an independent Melkite Greek Catholic Patriarchate. While previous studies have highlighted the crucial help that Sylvester received from the other Orthodox Patriarchs in Istanbul and Jerusalem in having his seat recognised by Ottoman authorities, the *ʿarżuḥāl*s examined in this paper showcase Sylvester’s own agency in negotiating Church affairs with the Porte. Either demanding imperial commands to reinforce his election as patriarch or to prevent Orthodox Christians from converting to Catholicism and imprisoning his rivals, Sylvester made full use of his position in the Ottoman administrative apparatus by using state-sanctioned practices to solve inter-confessional struggles. The paper argues that the Ottoman Empire provided a legal and administrative framework in which Orthodox Churches were not merely compelled to function under duress, but one which they found advantageous and from which they sought legitimisation for their own factional struggles.

## Plain Language summary

This article examines several petitions sent by Sylvester, Orthodox Patriarch of Antioch, to the Ottoman Imperial Council in Istanbul in the 1720s and 30s, showing how non-Muslims interacted with Muslim authorities to achieve their religious goals.

## Introduction

In 1724, with the election of two separate patriarchs, the once unitary Church of Antioch was effectively divided between the Orthodox and Catholic factions. Schism for some, communion with Rome for the others, the three-hundredth anniversary of this event was recently marked by a series of events and publications, which have the great merit of also bringing to attention the role played by the Ottoman Muslim authorities. ^1^ This article further explores how the Sublime Porte was involved in the quarrel between its Orthodox and Catholic subjects ( *reʿāyā*) by examining a set of original Ottoman-Turkish documents comprising petitions submitted early in his career by Sylvester, whom the Orthodox faction elected Patriarch of Antioch in 1724. These petitions described the problems the patriarch encountered during the first years of his reign, and called for the intervention of the Ottoman central government against his rival Seraphim Ṭanās, whom the pro-Catholic faction elected as Patriarch Cyril VI. Apart from the documents produced by Sylvester, this paper also includes a collective petition submitted in 1722 by three other Eastern patriarchs. All of the examined petitions are preserved as original documents in the Ottoman Archives in Istanbul, and their facsimiles, transliterations and translations are provided in the Appendices to this article. The analysis of these documents reveals that instead of being solely an oppressive “Turkish yoke”, ^2^ the Ottoman Empire provided a legal and administrative framework in which Orthodox Churches were not merely compelled to function under duress, but one which they found advantageous and from which they sought legitimisation for their own factional struggles.

The dichotomy between *Rūm* (Orthodox) and *Frenk* (Catholic) was a key concept in Sylvester’s petitions. Muslim Arabs first applied the term *Rūm* to the Romans, better known today as the Byzantines, whom they encountered in the seventh century, whereas *Frenk* can trace its roots back to the “Frankish” crusaders who invaded Syria and Palestine in the late eleventh century. With time, and especially after the Ottomans conquered the Eastern Mediterranean basin in the sixteenth century, the two terms gained a more religious meaning. Generally, *Rūm* were the Orthodox Christians, regardless of their ethnicity, although sometimes the term could take a narrower, ethnic meaning, that of “Greek” (thus *Rūmca* being the Greek language). ^3^ When more precise distinctions were needed, terms that combined both ethnicity and religion, such as “the Arab Orthodox community” ( *ʿArab Rūm tāʾifesi*) were used. ^4^ On the other hand, *Frenk* evolved from denoting Western Christians in general to Catholics, in particular. Although *Frenk* continued to be used as an umbrella term for Westerners, because of the Protestant Reformation, it gained a more pronounced religious meaning, denoting both foreigners and local Catholics. ^5^ Even if tax-paying Catholic *reʿāyā* were native to many places governed by the Ottomans, such as Bosnia, the Greek Archipelago or Galata, Catholicism could still be perceived as foreign. While the spiritual leaders of Eastern Christians, including the Greek Orthodox patriarchs, resided in Ottoman lands and submitted to the sultan, the pope was the independent ruler of a foreign polity, often on unfriendly terms with the Porte. The activity of many Catholic missionaries coming from lands outside of the “Well-Protected Domains” ( *Memālik-i Maḥrūse*), as the Ottomans called their realms, also contributed to this perceived “foreignness”, which Sylvester of Antioch denounced in his petitions.

Petitions represented the principal mechanism through which the Empire’s population communicated their grievances to Ottoman authorities, either orally or in writing. Those presented by government officials to higher authorities in the Imperial Council ( *Divān-ı Hümāyūn*) were known as *ʿarż*, whereas those submitted by lower-ranking subjects, including Christian clergymen, were known as *ʿarżuḥāl* (or *ʿarż-ı ḥāl* ). Petitions addressed to the sultan or grand vizier were heard several times a week in a dedicated building called the Chamber of Petitions ( *ʿarż odası*), located in the third court of the Topkapı Palace, behind the Gate of Felicity ( *bābü’s-sa ʿāde*). In theory, any Ottoman subject could write and submit their own petitions, ^6^ but by the eighteenth century, specially trained scribes ( *ʿarżuḥālcı*) were employed to redact *ʿarżuḥāl*s because of the strict drafting protocols that required a knowledge of both administrative and Islamic legal terminology and concepts. ^7^ Therefore, the written petitions examined in this article follow a standardised model consisting of several elements. ^8^

The petitions were written on the lower half of a large piece of paper, leaving much of the upper half blank, with only a simple invocation to God ( *daʿvet*), indicated through the Arabic third-person pronoun *hü* (lit. “He”). The first proper line of text of the *ʿarżuḥāl* consisted of a salutation to the official who was being addressed. If the addressee was the sultan, then he would be addressed as *pādişāh.* In contrast, lower-ranking officials, including grand viziers, would be addressed as *sultān*, to which several epithets such as “stately” ( *devletlü*) and “fortunate” ( *saʿādetlü*) were added, ending with the standard formula “May he be healthy!” ( *sağ olsun*). Since all petitions examined in this paper contain these latter elements, they were submitted to the grand vizier, and not the sultan. The demands for imperial commands ( *fermān*) and their preservation in the Ottoman Archives in Istanbul exclude the possibility that they were submitted to local authorities, such as the governors-general ( *beylerbeyi*) of Aleppo or Damascus.

The petitioner’s credentials came after this introductory protocol. While modest self-designations such as “this servant” ( *bu ḳūlları* or *bendeleri*) were used instead of names, the petitioner’s rank was a mandatory feature. Afterwards, the petitioner presented the focal point of the petitions, i.e. the problem(s) that required the Ottoman authorities’ intervention and then the request itself, which often materialised in the form of a *fermān.* The narrative exposition and request were separated by the phrase “it is requested that” ( *mercūdur ki*). The *ʿarżuḥāl* ended with another standardised formula through which the petitioner humbly declared that the decision lay solely with the addressee, followed by the petitioner’s signature and seal, usually placed on the back of the document directly over the signature. One must keep in mind that, at least in the documents analysed in this article, the “signatures” do not appear to have been written by the supplicants. The hand that wrote them is very similar to that of the main body of text, suggesting that it was the *ʿarżuḥālcı* who also “signed” the petitions. This is most clear in Document 3, where the signatures of all three patriarchs are clearly written in the same hand.

Unfortunately for researchers, most petitions are undated, so a significant amount of contextual information is needed to put them in historical perspective.

## Sylvester’s petitions

Sylvester submitted the first petition analysed in this paper, Document 1, ^9^ probably around 1725. Although undated, it was likely written soon after he was appointed Patriarch of Antioch and granted his diploma of appointment, or *berāt*, since the text mentions that the previous patriarch, Athanasios, had died, and now a *berāt* was issued for the new patriarch. This is the information that one would expect to find in a document issued shortly after Ottoman authorities confirmed the nomination of a new patriarch. Another clue for the dating of this petition lies on its reverse: Sylvester’s bilingual Greek – Ottoman-Turkish seal, which bears the year 1724 in Arabic figures. Thus, one may assume that the document was written in 1724. Although it would have been most convenient for historians if Sylvester, or other patriarchs, updated the year on their seals with regularity, evidence provided by another document in our analysis appears to contradict this theory. Instead, it would appear more likely that Sylvester’s seal, present on this and other documents, simply indicated the date of his appointment as Patriarch. ^10^ Similarly, Cyril VI’s seal also mentioned the year of his election, though this time according to the Hijri calendar. ^11^ Therefore, while it is safe to assume that Sylvester submitted this *ʿarżuḥāl* shortly after his appointment, the year, not to mention the month or day, is less certain.

The subject of Sylvester’s first petition to the Porte called for further confirmation of his ecclesiastical authority over the Patriarchate of Antioch. After his predecessor, Athanasios III Dabbās (in office 1685–1694, 1720–1724), died in August 1724, a rupture occurred within the Church of Antioch. A council of senior church hierarchs, headed by Jeremias III of Constantinople (in office 1716–1726, 1732–1733) and backed by the Grand Dragoman of the Porte, Gregory Ghika, ^12^ elected Sylvester, then a monk on Mount Athos, as the new Patriarch of Antioch. However, a group of pro-Catholic bishops who sought communion with Rome gathered in Damascus and elected their own candidate, Seraphim Ṭanās, who took the patriarchal name of Cyril VI. Thus, in late 1724, the Church of Antioch had two Patriarchs: Sylvester, who supported the Orthodox cause, and Cyril, who, shortly after his election, issued a Catholic profession of faith. ^13^ However, for their position to be officially recognised by the Ottoman authorities, they needed to secure a *berāt* from the sultan, a feat which, at this point, only Sylvester managed to achieve. With the support of the highest Greek-Orthodox officials in the Ottoman capital, as well as foreign diplomatic backing, ^14^ the sultan issued Sylvester’s *berāt* in August 1724. The opening protocol of Sylvester’s diploma of appointment is most interesting because, unlike other similar documents, it contained quite a detailed account of the conflict between the Orthodox and Catholic parties. ^15^

Nevertheless, securing a *berāt* did not mean that those who contested Sylvester accepted the situation, and they continued to champion Cyril. Therefore, through his *ʿarżuḥāl*, Sylvester asked the sultan to issue a corroborating *fermān* addressed to the Ottoman governors of Damascus, Aleppo, Sayda, and Tripoli – the most important cities of his diocese – so that they would respect the provisions of the *berat*, that is, to recognise Sylvester as the sole Patriarch of Antioch. The Grand Vizier Nevșehirli Damad Ibrahim Pasha ^16^ approved Sylvester’s request and ordered that the *berāt*’s provisions be respected. The requested *fermān* may have also acted as a safe conduct for the Patriarch, who travelled from Istanbul to Syria, the first visit of his diocese, in 1725–6. As he reached Aleppo in November 1725, ^17^ Sylvester must have submitted this petition before that date, while he was still in the imperial capital.

Our next document, or rather set of documents, presents a much more complex situation. On a single piece of paper, there is not one but three documents: two *ʿarżuḥāl*s and one *fermān.* ^18^ On the *recto* lies Document 2a, an *ʿarżuḥāl* signed by Sylvester, along with Document 2b, the copy of a *fermān* inscribed in the top right margin, or the *kenār*, while on the *verso* there is Document 2c, another *ʿarżuḥāl*, this time penned by the Patriarch Seraphim I of Constantinople ^19^ (not to be confused with Seraphim Ṭanās). The only dated document of the three is the *fermān*, which bears the date of *evāsıṭ-ı Rebiʿül-evvel* 1135, that is, 20–29 December 1722. Dating the two *ʿarżuḥāl*s, although being a rather simple task, cannot be done with utmost precision. This time, there is not one, but two patriarchal seals that may contain clues. Sylvester’s seal appears identical to the one on Document 1, analysed above, even mentioning the year 1724. While the other patriarch’s seal does not bear a date, Seraphim I of Constantinople is key to dating the two *ʿarżuḥāl*s, as he was in office only for a short time, between March 1733 and March or September 1734. ^20^ Therefore, the year 1724 on Sylvester’s seal(s) should not be considered a seal date, but rather a celebration of the year of his accession as Patriarch of Antioch. We can rather safely assume that Documents 2a and 2c were written sometime in 1733 or 1734, when Seraphim I was ecumenical patriarch, ^21^ while Document 2b served as a legal reference.

While the previously examined *ʿarżuḥāl* represented a simple request by Sylvester for a *fermān* to have his election recognised by local Ottoman officials in Syria, this set of three documents gives much more detail about the ongoing conflict between the Orthodox and Catholic factions. Having a Catholic rival elected by local clergy in Damascus, Sylvester’s fundamental mission was to convince the Ottoman central government that he was the rightful and sole patriarch of Antioch. Back in 1725, at a synod held in Constantinople, the highest representatives of the Orthodox Church from all over the Ottoman Empire condemned what they perceived to be Catholic heresies: the pope’s primacy, the Filioque, the existence of the Purgatory, whether to use leaven or unleaven bread during Holy Comunion, other issues such as what is allowed to eat during lent etc. ^22^ Sylvester, who attended this synod, would later publish an encyclical letter sent by the then-patriarch of Constantinople to Christians in Syria, along with other relevant texts condemning the Catholic faith, in a book printed in Bucharest in 1747. ^23^

But how could Sylvester convince the Ottoman authorities that he was the rightful patriarch and not his rival, Seraphim? The pope’s primacy could have raised suspicion, because the newly-converted Catholics would have officially recognised an often unfriendly foreign head of state as supreme leader. On the other hand, the Imperial Council would have probably not given too much attention to Christian theological debates, such as the relation between the three persons of the Trinity, or would have been much disturbed if Catholics had unleavened bread for communion. Therefore, Sylvester adopted a language that the Ottoman Muslim authorities knew and cared about: he insisted on the issue of political loyalty.

In his *ʿarżuḥāl* of 1733–1734, Sylvester explained how, in the past, some *Frenk* monks came to Ottoman Syria and began converting the local *Rūm* population. The vocabulary employed by the patriarch was as direct as it could be: Catholics used “perversion and corruption” ( *ıżlāl u ifsād*) ^24^ to turn the sultan’s non-Muslim subjects ( *ẕimmī reʿāyā*) from their old rites ( *ḳadīmī āyinleri*) and make them submit to “the Frankish sect” ( *Frenk meẕhebi*). He then went on to invoke a report made by some previous patriarchs concerning this affair, and mentions that a *fermān* had been issued to stop the conversions, but that it had never been put into practice. Sylvester even recalled here the names of the perpetrators: Euthymios, former metropolitan of Sayda, ^25^ and his followers and successors, Seraphim, who was referred to as Euthymios’s sister’s son ( *ḳız ḳarındaşı oğlı*), ^26^ and Father Gabriel, son of Finān ( *Baba Gavrīl veled-i Fīnān*). ^27^ Still in the past, another *fermān* was issued to condemn those troublemakers and to imprison them in a castle – this is Document 2b, the *fermān* that was copied on the margin ( *derkenār*) of Sylvester’s petition, to support the patriarch’s claims. However, this order, too, was not implemented.

Sylvester described the consequences of Seraphim’s and Gabriel’s actions bluntly: by becoming Catholic, the subjects renounced their subjecthood ( *reʿāyā reʿāyālıkdan çıḳub Frenk olmaları*). In other words, Sylvester argued that one could not be a subject of the sultan and a Catholic at the same time, and therefore converting to Catholicism meant treason. To make his point clear, Sylvester employed throughout his *ʿarżuḥāl* terms denoting mischief and social unrest, such as *fesād* (sedition), *ıżlāl* (perversion), and *ifsād* (corruption), thus hoping to convince the Imperial Council of the gravity of his rivals’ actions. Consequently, Sylvester asked the sultan for another *fermān* to have Seraphim and Gabriel apprehended on the spot, so they would not run away again, and then to exile and imprison them in the castle of Mağusa, ^28^ on the patriarch’s native island of Cyprus.

Document 2c, the other petition recorded on the reverse of this same piece of paper, signed by Patriarch Seraphim of Constantinople, largely reproduces Sylvester’s, in an effort to put more weight and credibility behind the request. Nevertheless, this document doubled down with a crucial argument, not specified in the other petitions: Cyril VI claimed the Patriarchate of Antioch despite not having received an investiture diploma ( *bilā berāt baṭriḳliḳ iddiʿā edüb*), and therefore, illegally. Doctrinal differences and conversions that led to social unrest aside, the ecumenical patriarch invoked the supreme instrument of legitimacy that a Christian hierarch had to obtain within Ottoman realms, the imperial *berāt* denoting the sultan’s official recognition. Thus, Seraphim of Constantinople took an extra measure of security. If Ottoman authorities did not consider the conversions unlawful, they could not deny the fact that Cyril VI illegally proclaimed himself patriarch of Antioch, since in the absence of a *berāt,* he was undermining the sultan’s authority. Another piece of indirect evidence denoting Seraphim’s baseless claim to the patriarchal throne, according to his Orthodox rivals, was that in these petitions he is never mentioned by his patriarchal name, Cyril.

The investigation into the petitions of high Orthodox clergy does not end here. Luckily, the Ottoman archives in Istanbul also preserve the original petition that Sylvester briefly mentioned as having been submitted by “the previous patriarchs” ( *selefim baṭriḳler*).

Document 3 is a collective *ʿarżuḥāl* signed by three Orthodox Patriarchs: Jeremias of Constantinople, Athanasios of Antioch (Sylvester’s predecessor), and Chrysantos Notaras of Jerusalem. ^29^ Unlike the other petitions, a scribe in the Imperial Council wrote down the date of its submission, 11 *Rebīʿül-evvel* 1135 (20 December 1722). It was known, from other sources, that the three patriarchs met in Istanbul in 1722 to discuss and condemn Catholic activities in Syria, ^30^ but, to my knowledge, scholars have never examined this document. Therefore, it represents a rare case in which three of the most senior Orthodox Church leaders in the Well-Protected Domains placed their signatures and seals on the same petition addressed to the Ottoman sultan.

This earlier *ʿarżuḥāl* detailed the troubles caused by the above-mentioned Euthymios, Seraphim, and Gabriel. While officially declaring to be *Rūm*, they nevertheless “submitted to the Franks’ religion” ( *Efrenc dīnine tebaʿiyyet*). Notice how, in comparison with Sylvester’s *ʿarżuḥāl*, where Catholicism was presented as a “sect” ( *meẕheb*), here it was described as a religion ( *dīn*), denoting that it was different from that of the *Rūm.* The petition then explained that the three troublemakers set out to convert the Orthodox subjects among the non-Muslims of Sayda ( *ehl-i ẕimmet Rūm reʿāyāsı*) and that they succeeded in converting most of them, except those living in the subdistrict ( *każā*) of Akka. When they failed to do so, we are told that they resorted to employing the now usual “perversion and corruption” ( *ıẓlāl* and *ifsād*), as well as committing murder ( *ḳatl* ). Seeking help, the threatened Orthodox subjects of Akka went to the Sharia court of Jerusalem, the seat of the larger district ( *sancaḳ*), where they obtained a favourable ruling. The former judge of Jerusalem, Mola Abdurrahman Efendi, along with other local Islamic scholars ( *müfti*) and the castellan ( *dizdār*) of Akka, decreed that the *Rūm*s should obey the petitioning patriarchs. Following these actions, the sultan issued a corroborating *fermān* for the imprisonment of Euthymios, who nevertheless managed to run away. Therefore, the problems persisted, and the three patriarchs complained that *Rūm ẕimmī*s, whether in Damascus, Jerusalem, Sayda or Akka, “were every day renouncing their status as subjects and submitting to the Franks’ religion” (Sylvester would use the same formulation in his *ʿarżuḥāl* some ten years later). Therefore, the three petitioning patriarchs asked for another imperial command to have Euthymios captured and imprisoned in the castle of Adana. It was this resulting *fermān* that was later recorded alongside Sylvester’s petition (Document 2b), and which the patriarch mentioned that it was never put into practice, thus asking again for the imprisonment of Euthymios’s followers (Euthymios had died in 1723), though that time in Mağusa, not in Adana.

Why did the Orthodox patriarchs choose the castles of Adana and Mağusa specifically to imprison their rivals? One possibility could be that Sylvester, a native of Cyprus, kept close relations with Ottoman officials on the island. ^31^ As for Adana, also under the ecclesiastical jurisdiction of Antioch, it seems that the castle there was a preferred destination for *zimmi* troublemakers, as attested in a *fermān* from 1703, through which Athanasios Dabbās, then Metropolitan of Aleppo, obtained the release of one of his servants. ^32^ In contrast, for example, it is a known fact that the governor of Damascus, Osman Pasha, supported Cyril VI, ^33^ so one could not have expected him to diligently execute the *fermān* and detain the culprits. Nevertheless, this remains for the moment only a working hypothesis, and more research is required before formulating any conclusions.

In any case, these petitions failed to achieve their goal of imprisoning the main leaders of the pro-Catholic faction, despite the support obtained from the Imperial capital and the successive *fermān*s. In 1725, the *ḳapıcıbaşı* sent to apprehend Seraphim and the other pro-Catholic leaders returned only with Gerasimos, Bishop of Aleppo. ^34^ Cyril VI found refuge in the Mountains of Lebanon, where a strong Greek Catholic community would develop. ^35^

After the Patrona Halil rebellion of 1730, which saw the dethronement of Sultan Ahmed III, Cyril VI hoped to gain recognition from the new sultan, Mahmud I. In the following years, the French ambassador in Constantinople tried to obtain a *berāt* from the sultan on his behalf. However, Sylvester once more outbested Cyril, as he had his own *berāt* renewed by Mahmud on 2 October 1730, immediately after the sultan’s enthronement. In 1733, the Propaganda Fide declared Cyril patriarch, although the pope would only issue him a *pallium* in 1744. ^36^ Sylvester’s *ʿarżuḥāl* of 1733 or 1734 must have appeared in this context in which Cyrill VI resumed his contentious actions.

Another important episode of this rivalry over the Patriarchate of Antioch occurred in 1745 when, for a few months, the sultan recognised Cyril VI as patriarch of Antioch, by issuing him a *berāt.* However, Sylvester obtained his deposition only six months later, in August. Essential to the topic of this paper, Cyril’s *berāt* titled him the *Rūm* Patriarch of Antioch. ^37^ It appears that the rhetoric earlier employed by Sylvester and other Orthodox patriarchs was still relevant in the middle of the eighteenth century, as it seemed very difficult, if not impossible, for the Porte to acknowledge a *Frenk* leading the Church of Antioch. In his own petitions addressed to the Imperial Council, Cyrill argued that Sylvester was not suitable to be patriarch because he did not have the support of Aleppo’s *Rūm* subjects. ^38^

Nevertheless, even though Cyril enjoyed a very brief recognition by the Ottoman authorities, he is considered the founding patriarch of the Melkite Greek Catholic Church of Antioch to this day.

## Conclusions

In conclusion, the documents examined in this paper showcase a new dimension of the struggle between the Catholic and Orthodox factions in the early eighteenth-century Ottoman Empire. We can now hear the voices of Sylvester and other Eastern Patriarchs employing the administrative language of the Porte, though most likely filtered by specialised scribes. These hierarchs presented the interreligious struggle in a way that the Ottoman administration could understand and, more importantly, care about. They knew “which buttons to press” in order to obtain the desired actions against their Catholic rivals.

By tying subjecthood to Orthodoxy, Sylvester and other Eastern Patriarchs were essentially seeking to outlaw conversions to Catholicism. Unlike apostasy from Islam, which was considered a capital offence under the Sharia, non-Muslims were not prohibited from changing their religion and, at least in theory, Muslim authorities should not have been bothered if Christians moved from one Church to another, as the Porte decreed several times that “unbelief represented a single nation” ( *kefere ṭāʾifesi millet-i vaḥide*). ^39^ Thus, facing ever-increasing Catholic conversions among their flock, Christian Orthodox patriarchs effectively strived to counter the permissivity of Islamic Law regarding the conversion of non-Muslims by equating Catholicism with sedition and corruption. Similar to apostasy, offences against public order and rebellion against the sultan were considered capital offences. ^40^

Of course, the Porte had for centuries ruled over many Catholic communities, who were, for the most part, paying their taxes and conforming to their status as *ẕimmī*s. *Berāt*s issued to Catholic bishops in the Aegean, for example, did not refrain from mentioning the *Frenk* denomination. ^41^ What had started to change, by the early eighteenth century, was indeed the foreign interference. Abusing capitulatory stipulations, foreign powers (though not only Catholic ones), were beginning to extend their protection over Ottoman subjects. Later in the century and especially in the nineteenth century, *berātlı*s would form a social class of their own. These were Ottoman subjects who were exempted from various taxes because of their employment by foreign diplomatic and consular missions. Although adopting the European protecting Power’s religious confession was not a mandatory condition, many Eastern Christians used conversion as a fast track to becoming *berātlı.* ^42^ An Ottoman Catholic was better positioned to become a protégé of a Catholic power like France, for example.

The petitions discussed in this paper support the need to move past the idea that the Ottoman Empire represented a purely oppressive polity for the Christians under its rule. Feeling threatened by the spread of Catholicism, Orthodox hierarchs sought protection from their Muslim overlords, to whom they presented themselves as loyal subjects, and made full use of the legal levers of the empire to support their cause. They went to local Islamic courts and submitted *ʿarżuḥāl*s to the Imperial Council in Istanbul to have their Christian rivals imprisoned. Sylvester, while not the first one to employ these methods, was especially interested in having the Ottoman administration on his side if he was to prevail against his Catholic rival, Cyril VI. And considering that he managed to remain patriarch for more than forty years, until his death in 1766, albeit with a brief interruption in 1745, one may say that he was successful in doing so.

## Ethics and consent

Ethical approval and consent were not required.

## Data Availability

Zenodo. The ʿarżuḥāls of Sylvester, Patriarch of Antioch: Negotiating Church Affairs with the Sublime Porte in the First Half of the 18th Century. https://doi.org/10.5281/zenodo.19128739. (Dipratu, R. 2026). This project contains the following underlying data:
• APPENDICES Sylvester arzuhals.docx: Facsimiles, transliterations and translations of the archival documents analysed in this paper Data is available under the terms of the Creative Commons Attribution 4.0 International.

## Footnotes

^1^
Rami Wakim, Charbel Nassif (eds.), *Jubilé et Anamnèse. Recueil d’articles à l’occasion du tricentenaire du rétablissement de l’union ecclésiastique entre l’Église melkite et l’Église de Rome* (Éditions du Patriarcat grec melkite catholique, 2024); Bernard Heyberger, Željko Paša, Ronney el Gemayel (eds.), *La division de l’Église rūm d’Antioche en 1724 et la construction des identités confessionnelles orthodoxes et catholiques* (CEDRAC, PIO, 2024); Archim. Jacob Khalīl (ed.), *Al-Kanīsa al-Ūrthūdhuksīya min al-qarn al-khāmis ʻashar ilā al-qarn al-thāmin ʻashar. Naḥwa fahm daqīq li-l-tārīkh* (St. John of Damascus Institute of Theology, University of Balamand, 2025).

^2^
Dariusz Kołodziejczyk, “The ‘Turkish Yoke’ Revisited: Non-Muslim Subjects Between Loyalty, Alienation, and Riot,” *Acta Poloniae Historica* 93 (2006): 177–195.

^3^
Colin E. Bosworth, “Rūm,” in *Encyclopaedia of Islam New Edition*, vol. 8, ed. by C.E. Bosworth, E. van Donzel, W.P. Heinrichs and G. Lecomte (Brill, 1995), 601–6; Cemal Kafadar, “A Rome of One’s Own: Reflections on Cultural Geography and Identity in the Lands of Rum,” *Muqarnas* 24 (2007): 7–25.

^4^
Hasan Çolak, “Relations Between the Ottoman Central Administration and the Greek Orthodox Patriarchates of Antioch, Jerusalem and Alexandria: 16 ^th^ – 18 ^th^ Centuries” (PhD diss., The University of Birmingham, 2012), 154–7.

^5^
Eva Johanna Holmberg, “In the Company of Franks: British Identifications in the Early Modern Levant c. 1600,” *Studies in Travel Writing* 16, no. 4 (2012): 363–74; Elif Bayraktar-Tellan, “The Clash of ‘ *Rum’* and ‘ *Frenk’*: Orthodox-Catholic Interactions in the Aegean Islands in the Mid-Seventeenth to Mid-Eighteenth Centuries and their Impact in the Ottoman Capital,” in *Islands of the Eastern Mediterranean. A History of Cross-Cultural Encounters*, ed. by Özlem Çaykent and Luca Zavagno (I.B. Tauris, 2014), 64–78; Ioana Feodorov, “Les *Firanğ* – Francs, Européens ou catholiques? Témoignage d’un chrétien syrien du XVIIe siècle,” *Orientalia Christiana Periodica* 82 (2016): 179–210.

^6^
Foreign merchants, on the other hand, had to appeal to their consul or ambassador, who then submitted a petition on their behalf. See Tommaso Stefini, “Ottoman Justice and Political Economy of Empires: Venetian Merchants in the Seventeenth Century,” *Comparative Studies in Society and History* (2025): 14–15.

^7^
This may be the explanation why in Document 2a (see the transliteration in the Appendices), the names of Seraphim Ṭanās and Gabriel b. Finān are first written correctly, but later misspelt.

^8^
Mübahat S. Kütükoğlu, *Osmanlı Belgerlerinin Dili (Diplomatik)* (Kubbealtı Akademisi Kültür ve San’at Vakfı, 1994): 217–9; Mehmet İpşirli, “Arzuhal”, in *İslam Ansiklopedisi*, vol. 3 (TDV İslâm Araştırmaları Merkezi, 1991), 447–8; Aysema Pelin Şaşmaz, “Klasik Dönem Osmanlı Devleti’nde Halkın Adalete Ulaşma Aracı Olarak ‘Arz-I Hal’ Uygulaması”, *Uluslararası Yönetim Akademisi Dergisi* 6, no. 4 (2023): 1243–56.

^9^
BOA, C.ADL. 73, 4405.

^10^
In Ottoman documents, Sylvester used a seal that contained only text, whereas on other documents, he used another, larger seal that also featured an image of St. Peter. For this and other discussions over Sylvester’s (and Cyril VI’s) seals, see Mihai Țipău, *Sylvester of Antioch. Life and Achievements of an 18th-Century Christian Orthodox Patriarch* (De Gruyter, 2025), 235, 247–8, 415 (facsimile).

^11^
Hasan Çolak, “When a Catholic is Invested as the Orthodox Patriarch of Antioch: Serafeim/Kyrillos Tanas and the Ottoman Central Administration in 1745,” *Collectanea Christiana Orientalia* 20 (2023): 42, 54 (facsimile).

^12^
Çolak, “Relations,” 183.

^13^
Çolak, “Relations,” 185.

^14^
Carsten-Michael Walbiner, “The Split of the Greek Orthodox Patriarchate of Antioch (1724) and the Emergence of a New Identity in Bilad al-Sham as Reflected by Some Melkite Historians of the 18th and early 20th centuries,” *Chronos* 7 (2003): 18.

^15^
Hasan Çolak, Elif Bayraktar-Tellan, *The Orthodox Church as an Ottoman Institution. A Study of Early Modern Patriarchal Berats* (The Isis Press, 2019), 92–3, 218–20.

^16^
Ibrahim Pasha and Sylvester also had a common interest regarding book printing. For the grand vizier’s role in establishing the first Turkish printing press in the Ottoman capital see Orlin Sabev, *The Müteferrika Press. Arabic Typography in an Ottoman Context* (De Gruyter, 2025), 48–51, 54–8; for Sylvester’s printing activity see Țipău, *Sylvester of Antioch*, 173–239.

^17^
Țipău, *Sylvester of Antioch*, 57, 164.

^18^
BOA, C.ADL. 35, 2074.

^19^
Throughout these Ottoman-Turkish petitions, including their signatures, the ecumenical patriarchs mention their see as *İstanbul.* However, the Ottoman-Turkish text of their seals referred to it as *Ḳosṭanṭiniyye*, the same term used for the place of issue in imperial *fermān*s. Since the term refers to the Church of Constantinople, I will use “Patriarch of Constantinople” throughout this article.

^20^
Demetrius Kiminas, *The Ecumenical Patriarchate. A History of Its Metropolitans with Annotated Hierarch Catalogs* (Borgo Press, 2009), 41.

^21^
There was another Patriarch of Constantinople called Seraphim during Sylvester’s reign, Seraphim II, but he ruled much later, between 1757 and 1761 (Kiminas, *Ecumenical Patriarchate*, 41). However, at this later date, the events described in the three documents were no longer relevant.

^22^
Țipău, *Sylvester of Antioch*, 53.

^23^
Ioana Feodorov, “Sylvester of Antioch’s Arabic Books Printed in 1747 at Bucharest. Recent Findings,” *Scrinium* 19 (2023): 18–24 (5–27).

^24^
A contemporary English merchant and supporter of Sylvester used similar terminology in describing the pro-Catholic faction in Syria: “the Missionaries of Rome, and their perverted Proselytes”, “the Pervertiti”: Simon Mills, *A Commerce of Knowledge. Trade, Religion, and Scholarship Between England and the Ottoman Empire, c. 1600–1760* (Oxford University Press, 2020), 241, and Țipău, *Sylvester of Antioch*, 83.

^25^
After the second appointment of Athanasius III Dabbās as Patriarch in 1720, Euthymios Sayfi (d. 1723) became the leader of the pro-Catholic faction within the Church of Antioch. Walbiner, “The Split,” 11–13; Țipău, *Sylvester of Antioch*, 5–11, 15.

^26^
Bernard Heyberger, *Les chrétiens du Proche-Orient au temps de la Réforme catholique (Syrie, Liban, Palestine, XVIIe-XVIIIe siècles)* (École française de Rome, 1994), 121. Sylvester also seems to have been the nephew of the preceding patriarch, Athanasios. See Țipău, *Sylvester of Antioch*, 34.

^27^
The pro-Catholic priest Gabriel b. Finān, one of the principal supporters of Euthymios Sayfi, was consecrated as Bishop of Banyas (Caesarea Philippi) in 1724, taking the name of Basil. The same year, he was one of the three bishops who elected Cyril VI as Patriarch of Antioch. Abdallah Raheb, “Greek Melkite Catholic Patriarchate of Antioch: Birth, Evolution, and Current Orientation,” translated by Nicholas J. Samra, 9 (1–21) https://phoenicia.org/pdfs/melkite_greek_catholic.pdf (accessed 9 February 2025) (originally published in *Ekklesiastikos Pharos* 52, nos. 2–3, 1969); Asad Rustum, *Kanisat Madinat Allah Antakya al-’Uzma* [The Church of the Great City of God Antioch], vol. 3 (Editions St. Paul, 1988), 135–141, translated by Samuel Noble on https://araborthodoxy.blogspot.com/2024/07/asad-rustom-on-patriarch-athanasius-iii_29.html (accessed 9 February 2025); Çolak, “Relations,” 180–1; Țipău, *Sylvester of Antioch*, 48.

^28^
Today known in Turkish as Gazimağusa, in Greek Αμμόχωστος, and historically as Famagusta.

^29^
BOA, AE.SAMD.III 211, 20436.

^30^
Țipău, *Sylvester of Antioch*, 7.

^31^
Another Christian troublemaker imprisoned in Mağusa appears in a later *ʿarżuḥāl* of Anthimos, Patriarch of Antioch, submitted on 29 *Ṣafar* 1208 (6 October 1793). C.ADL. 14, 90.

^32^
BOA, A.DVNS.MHM.d 113, p. 43, no. 167. Also see Cesare Santus, “A Triangular Relationship: the Role of Eastern Patriarchates, Rome, and Ottoman Authorities in Shaping the Christian Communities of the Empire,” in *La division de l’Église rūm d’Antioche*, ed. by B. Heyberger, Z. Paša and R. el Gemayel (CEDRAC, PIO, 2024), 99.

^33^
Osman Pasha had previously also secured the freedom of Euthymios Sayfi, but would be later deposed because of his support to Cyril VI. See Heyberger, *Les chrétiens du Proche-Orient *, 122, 126; Țipǎu, *Sylvester of Antioch*, 15, 48, 53.

^34^
Çolak, “Relations,” 173; Țipǎu, *Sylvester of Antioch*, 53.

^35^
Aurélien Girard, “Une Église aux mains des moines? Chouérites et salvatoriens dans l’Église melkite rūm-catholique au XVIIIe siècle,” in *La division de l’Église rūm d’Antioche*, ed. by B. Heyberger, Z. Paša and R. el Gemayel (CEDRAC, PIO, 2024), 275–327.

^36^
Çolak, “When a Catholic,” 34; Țipău, *Sylvester of Antioch*, 82–4.

^37^
Çolak, “When a Catholic,” 50.

^38^
Çolak, “Speaking the Ottoman Language: The Schism of 1724 and the Ottoman Central Administration,” in *La division de l’Église rūm d’Antioche*, ed. by B. Heyberger, Z. Paša and R. el Gemayel (CEDRAC, PIO, 2024),122–3.

^39^
For the use of this phrase in *ʿahdnāme*s, or capitulations, see Viorel Panaite, *Ottoman Law of War and Peace. The Ottoman Empire and Its Tribute-Payers from the North of the Danube*, second revised edition (Brill, 2019), 42; Stefini, “Ottoman Justices,” 16; for its implementantion in *fermān*s see Oded Peri, *Christianity under Islam in Jerusalem. The Question of the Holy Sites in Early Ottoman Times* (Brill, 2001), 155; Daniel Goffman, “Ottoman Millets in the Early Seventeenth Century,” *New Perspectives on Turkey* 11 (1994): 139–40.

^40^
Uriel Heyd, *Studies in Old Ottoman Criminal Law*, ed. by V.L. Ménage (Clarendon Press, 1973), 261–2. See also Palmira Brummet, “Classifying Ottoman Mutiny: The Act and Vision of Rebellion,” *Turkish Studies Association Bulletin* 22, no. 1 (Spring 1998): 91–107.

^41^
Radu Dipratu, “Ottoman Attitudes Towards the Re-establishment of the Catholic See of Izmir (Smyrna) in the First Half of the Seventeenth Century,“in *Latin and Eastern Catholicism in Ottoman Anatolia. Social, Economic, and Religious Inquiries from 14 ^th^–20 ^th^ Centuries*, ed. by V. R. de Obaldía, R. Dipratu, A. Massot, and P. Rohan (The Isis Press, 2025), 301–34.

^42^
Maurits van den Boogert, *The Capitulations and the Ottoman Legal System. Qadis, Consuls and* Beratlıs *, in the 18 ^th^ Century* (Brill, 2005), 101–2. For the interesting case of an Aleppine Maronite who tested the limits of this protection system see Paul Duvivier’s forthcoming article “L’affaire Karaly – La protection au Levant: enjeux et débats autour des berats à Alep au XVIIIe siècle.”
