# Supplementary material for: The ʿarżuḥāls of Sylvester, Patriarch of Antioch: Negotiating church affairs with the Sublime Porte in the first half of the 18th Century
Source: Open Res Eur. 2026 Aug 8;6:132. Originally published 2026 Apr 22. [Version 2] doi: 10.12688/openreseurope.23367.2 (PMC13560867; doi:10.12688/openreseurope.23367.2)
Supplement: Peer review history

# Reviewer report: Reviewer response for version 2

**DOI:** 10.21956/openreseurope.26802.r78805

**Published:** 2026-08-13

**Authors:** Michele Tantardini

**Affiliations:** 1 Penn State Harrisburg, Middletown, Pennsylvania, USA

I thank the author for addressing the suggestions I provided during the first round of review. I appreciate their careful consideration of my comments and the revisions made to the manuscript. I have no further comments or concerns and am satisfied with the current version.

If any, are all the source data and materials underlying the results available?

Yes

Is the work original in terms of material and argument?

Yes

Is the work clearly and cogently presented?

Partly

Is the argument persuasive and supported by evidence?

Partly

Does the research article contribute to the cultural, historical, social understanding of the field?

Partly

Does it sufficiently engage with relevant methodologies and secondary literature on the topic?

Partly

Reviewer Expertise:

Religion, faith, spirituality, and Public Administration.

I confirm that I have read this submission and believe that I have an appropriate level of expertise to confirm that it is of an acceptable scientific standard.

# Reviewer report: Reviewer response for version 2

**DOI:** 10.21956/openreseurope.26802.r78804

**Published:** 2026-08-13

**Authors:** Fatma Sel Turhan

**Affiliations:** 1 Sociology, Istanbul Technical University, Istanbul, İstanbul, Turkey

The author had addressed the concerns I raised in my peer review report appropriately.

If any, are all the source data and materials underlying the results available?

Yes

Is the work original in terms of material and argument?

Yes

Is the work clearly and cogently presented?

Partly

Is the argument persuasive and supported by evidence?

Partly

Does the research article contribute to the cultural, historical, social understanding of the field?

Yes

Does it sufficiently engage with relevant methodologies and secondary literature on the topic?

Yes

Reviewer Expertise:

Eighteenth and nineteenth century Ottoman Empire, Ottoman Christians, state-church relations, multiculturalism.

I confirm that I have read this submission and believe that I have an appropriate level of expertise to confirm that it is of an acceptable scientific standard.

# Reviewer report: Reviewer response for version 1

**DOI:** 10.21956/openreseurope.25280.r73035

**Published:** 2026-06-01

**Authors:** Fatma Sel Turhan

**Affiliations:** 1 Sociology, Istanbul Technical University, Istanbul, İstanbul, Turkey

This is a well-written article that addresses an original topic and is supported by archival material and relevant secondary sources. However, to further enrich the article and strengthen its arguments, the following suggestions are made to the author:

 1) The information in the first paragraph on page four (the section beginning ‘The petitions were written’) is taken from Mübahat Kütükoğlu’s book, but the author has not included a footnote. This paragraph requires a footnote.

 2) In the section on 'Sylvester's petitions', the first document does not include a date; however, the State Archives provide the date 14 Rabiulahir 1137 AH, which corresponds to 31 December 1724. This date likely originates from the file or register in which the document was first found, and it serves to support the author’s rough estimate of 'around 1725'. Mentioning that this date is cited in the catalogue (even if not in the archive document itself) and providing an explanation of the probable reasons for its inclusion would strengthen the author’s claim.

 3) In the first paragraph on page 6, the author asks: ‘But how could Sylvester convince the Ottoman authorities that he was the rightful patriarch and not his rival, Seraphim?’ However, within the Ottoman Empire, the Patriarchate of Constantinople held the authority to appoint officials under its jurisdiction. In accordance with this authority, the Patriarchate could appoint regional patriarchs operating within Ottoman territories and notify the relevant authorities of their names. The authorities then issue a charter to the relevant person. As Sylvester had been elected by the Patriarchate of Constantinople, the Ottoman authorities had no reason to doubt his status as Patriarch of Antioch. Rather than convincing the Ottoman authorities of his legitimacy as patriarch, the issue here is a request for the state to intervene in the efforts of Catholic missionaries in Ottoman Syria to convert the Orthodox population to Catholicism. Given that the Orthodox Church in Istanbul was an officially recognised church, it is not surprising that it would make such a demand. Therefore, rather than focusing on how Sylvester would convince the Ottoman authorities, the author should consider the circumstances in which Seraphim made this claim and its link to missionary activities that accelerated during this period.

 4) In the third paragraph on page 7 (the section beginning with ‘This earlier’), the use of the term ‘din’ instead of ‘mezhep’ is cited as a way of emphasizing the distinction from the Greeks. However, this is a general usage and is also applied to Catholics operating with the Ottoman Empire’s official permission, such as the Bosnian Catholics. Although not as widespread as the concepts of ‘millet’ and ‘mezhep’, ‘din’ is also a term used to define denominational differences. (See, for example, Cevdet Adliye 21/1289)

 5)    The author mentions ‘foreign interference’ in the concluding section regarding efforts to convert the growing number of Ottoman Orthodox Christians to the Catholic denomination during this period; however, there is no prior reference to this. If the missionary activities and efforts to convert the Orthodox population to the Catholic denomination, which increased in the Ottoman territories at the beginning of the eighteenth century, were analysed in greater detail —taking into account the Ottoman Empire’s relations with the states sponsoring these missionary activities— this would significantly strengthen the article. If this topic were not merely addressed in the conclusion but incorporated into the main framework of the article, it would make a significant contribution to historical writing. In this context, it would be beneficial to include State Archives of Turkey, Ottoman Archives, Cevdet Adliye 59/3570 in the study. Clearly, the increasing efforts to convert people to Catholicism during this period were not limited to the Orthodox Church. The Armenian Patriarchate also had similar complaints (see Ottoman Archives, İE. DH 34/2955), so the fact that the two officially recognised churches lodged similar complaints with the authorities provides information about the increasing missionary activities of this period, as well as the relationship between the two churches and the state itself.

If any, are all the source data and materials underlying the results available?

Yes

Is the work original in terms of material and argument?

Yes

Is the work clearly and cogently presented?

Partly

Is the argument persuasive and supported by evidence?

Partly

Does the research article contribute to the cultural, historical, social understanding of the field?

Yes

Does it sufficiently engage with relevant methodologies and secondary literature on the topic?

Yes

Reviewer Expertise:

Eighteenth and nineteenth century Ottoman Empire, Ottoman Christians, state-church relations, multiculturalism.

I confirm that I have read this submission and believe that I have an appropriate level of expertise to confirm that it is of an acceptable scientific standard, however I have significant reservations, as outlined above.

# Response

**Published:** 2026-07-15

**Authors:** Radu Dipratu

Thank you for your careful read of my article and your very valuable insights! I implemented some of them in the revised version and I will briefly answer your five points here: 

1. Indeed, I took the information from Kütükoğlu and other authors, but I did reference them in the previous footnote. After reading your comment, I moved the footnote at the end of this paragraph for better visibility. 
2. I refrained from mentioning the dates given by BOA since it is not always clear where did they take them from, and sometimes they are simply wrong. However, after your suggestion, I added footnotes for all three documents mentioning the dates given by BOA. 
3. The principal issue was that there were two people elected as Patriarch of Antioch and both of them seeked official recognition from the Porte; which, at this time, could only recognize one Patriarch. Therefore, I think my question is valid, since it was crucial both for Sylvester and Seraphim/Cyrill to convince the Porte that only one of them was the rightful patriarch. 
4. I did not claim that using “din” was unusual, just that it had a slightly different meaning than “mezheb”. 
5. The topic of foreign Catholic missionaries has seen its fair share of studies, and I quote some of them in the footnotes. Thank you for mentioning those documents, but unfortunately, I do not have access to them and, in any case, discussing more about foreign missionaries or their conversions of Armenians would diverge from the scope of this article, which is centered of Church of Antioch in a very specific timeframe.

# Reviewer report: Reviewer response for version 1

**DOI:** 10.21956/openreseurope.25280.r73034

**Published:** 2026-05-06

**Authors:** Michele Tantardini

**Affiliations:** 1 Penn State Harrisburg, Middletown, Pennsylvania, USA

Overall, this contribution is promising and well-documented, based on a valuable collection of Ottoman archival petitions related to Sylvester, Patriarch of Antioch. It effectively argues that Orthodox hierarchs could strategically leverage Ottoman administrative mechanisms. To strengthen the argument and enhance clarity, I recommend:

 1) including a detailed methodology section explaining not only what arżuḥāl are but also how the documents were analyzed: such as the criteria for selecting these documents over others (representativeness of the sample) and the specific analytical methods used (e.g., close reading, thematic coding).

 2) providing a clearer and potentially earlier context for Ottoman governance of non-Muslims and inter-confessional conflicts, as the conclusions depend on how the Porte, provincial officials, courts, berats, and fermans shaped options for Orthodox and Catholic actors.

If any, are all the source data and materials underlying the results available?

Yes

Is the work original in terms of material and argument?

Yes

Is the work clearly and cogently presented?

Partly

Is the argument persuasive and supported by evidence?

Partly

Does the research article contribute to the cultural, historical, social understanding of the field?

Partly

Does it sufficiently engage with relevant methodologies and secondary literature on the topic?

Partly

Reviewer Expertise:

Religion, faith, spirituality, and Public Administration.

I confirm that I have read this submission and believe that I have an appropriate level of expertise to confirm that it is of an acceptable scientific standard, however I have significant reservations, as outlined above.

# Response

**Published:** 2026-07-28

**Authors:** Radu Dipratu

Thank you for your observations! I have added an additional paragraph to the Introduction in which I briefly discuss the historical context of Ottoman administration over non-Muslim communities, as well as a couple of paragraphs detailing the methodology.
